# Factors Influencing Loss of Independence among Older Adults : Race/Ethnicity as a Moderator in NHATS

**DOI:** 10.1093/geroni/igaf122.3359

**Published:** 2025-12-31

**Authors:** Young-Shin Lee, Leticia Camacho, Hee-Jin Jun

**Affiliations:** San Diego State University, San Diego, California, United States; San Diego State University, San Diego, California, United States; San Diego State University, San Diego, California, United States

## Abstract

Disability in later life is a significant contributor to loss of independence (LoI). Despite its complexity, late-life disability remains underexplored. This study investigates factors influencing LoI across ethnic/racial groups, using the International Classification of Functioning, Disability, and Health model. Using 2022 National Health and Aging Trends Study data, LoI in older adults was assessed using the Vulnerable Elders-13 Survey (VES: 0–10), where higher scores denote increased vulnerability. Risk factors included body function, physical performance, and social participation; and gender, ethnicity, health issues, and environmental factors as covariates. Multiple regression analyses examined association with LoI and interaction effect among body function, performance, and social participation. Among 7,106 older adults, 42% were at high risk of LoI. Physical performance and limited social participation significantly interacted with race/ethnicity. Stratified analyses showed that physical performance was most strongly associated with LoI among White group (β = -0.42), followed by Black (β = -0.34) and Hispanic adults (β = -0.33). Among Black group, health conditions (β = 0.54) had the strongest impact on LoI, compared to White (β = 0.30) or Hispanic (β = 0.34) as well as restricted social participation (β = 0.804) on LoI, followed by Hispanic (β = 0.49) and White (β = 0.46) group. Limited English proficiency was a significant risk factor of LoI only for Hispanics (β = 0.57). Findings highlight that while physical function and performance are consistent predictors of LoI, the influence of health conditions, social participation, and language barriers differs across racial/ethnic groups.

